# Care-Seeking and Management of Common Childhood Illnesses in Tanzania – Results from the 2010 Demographic and Health Survey

**DOI:** 10.1371/journal.pone.0058789

**Published:** 2013-03-12

**Authors:** Catherine Kahabuka, Gunnar Kvåle, Sven Gudmund Hinderaker

**Affiliations:** Centre for International Health, Faculty of Medicine and Dentistry, University of Bergen, Bergen, Norway; Kenya Medical Research Institute - Wellcome Trust Research Programme, Kenya

## Abstract

**Background:**

Malaria, pneumonia and diarrhoea continue to kill millions of children in Africa despite the available and effective treatments. Correct diagnosis and prompt treatment with effective drugs at the first option consulted for child care is crucial for preventing severe disease and death from these illnesses. Using the 2010 Demographic and Health Survey data, the present study aims to assess care-seeking and management of suspected malaria, pneumonia and diarrhoea at various health care facilities in Tanzania.

**Methods:**

We analyzed data for 8176 children born within a 5 years period preceding the survey.The information was collected by interviewing 5519 women aged 15–49 years in 10,300 households selected from 475 sample points throughout Tanzania.

**Results:**

The most common first option for child care was PHC facilities (54.8%), followed by private pharmacies (23.4%). These were more commonly utilized in rural compared to urban areas: 61.2% versus 34.5% for PHC facilities, and 26.5% versus 17.7% for pharmacies. Women in urban areas and those with higher level of education more commonly utilized higher level hospitals and private facilities as their first option for child care. Only one in four children with fever had received a blood test during the illness with lowest proportion being reported among children solely attended at PHC facilities. Use of abandoned antimalarial drugs for the treatment of suspected malaria was also observed in public health facilities and antibiotics use for diarrhoea treatment was high (49.0%).

**Conclusions:**

PHC facilities and pharmacies most commonly provided sub-optimal care. These facilities were more commonly utilized as the first option for child care in rural areas and among the poor and non-educated families. These are groups with the highest child mortality, which calls for interventions’ targeting improvement of care at these facilities to further reduce child mortality from treatable illnesses in Tanzania.

## Introduction

Even though the number of deaths among children under the age of five has fallen globally, still one in eight children dies before age five in Sub-Saharan Africa [1]. Despite the available and effective treatments, pneumonia, diarrhoea and malaria remain the biggest killers of underfive children globally, accounting for 18%, 15% and 8% of all child deaths respectively in 2008 [2]. The three diseases were responsible for more than half of all child deaths in the African region in 2008; pneumonia 18%, diarrhoea 19% and malaria 16% [3]. Primary prevention of these infections is often difficult to achieve, particularly for children from poor families, hence correct diagnosis and prompt treatment with effective drugs very crucial in preventing child deaths from these illnesses.

Primary health care (PHC) facilities, i.e. dispensaries and health centres, are the closest and most often the first contact for sick children when the disease is still mild. Proper case management at this level of care is critical in order to prevent severe disease and deaths from these illnesses. However, several studies in Sub-Saharan Africa have reported poor quality of services, including mismanagement of sick children, at this level of care [4], [5], [6]. Other studies have reported bypassing of this level for child care in preference for higher level hospitals which are believed to provide better services [7], [8], [9].

A significant reduction in child mortality have been observed in Tanzania during recent years [10]. However, according to the 2010 Tanzania Demographic and Health Survey (TDHS) report, still one out of 20 children dies before their first birthday, and one out of 12 before their fifth birthday [11]. Malaria, pneumonia and diarrhoea as well account for the majority of child deaths in Tanzania [11]. Tanzania has a high density of PHC facilities. By the year 1992, about 72% of the Tanzanian population was reported to live within 5 km of a health facility [12]. This proportion was increased to about 90% in 2007 [13]. According to Tanzanian referral system, PHC facilities are supposed to be the first contact for mild conditions. However, several studies have documented poor quality of services at most of these facilities [14], [15], [16], [17], [18], causing some of the care-seekers to bypass them while seeking child care [19], [20], [21].

Using the most recent national survey data the current study aims to assess 1) the utilization of PHC facilities as the first option for child care in relation to care seekers’ background characteristics, and 2) the management of the common childhood conditions above (malaria, pneumonia and diarrhoea) at various types of health care facilities consulted for child care in Tanzania.

## Materials and Methods

Our study utilized data from the 2010 Tanzania DHS, which is the eighth and most recent in a series of national sample surveys that measure levels, patterns, and trends of demographic and health indicators in Tanzania. DHS are nationally-representative cross-sectional household surveys (involving between 5,000 and 30,000 households) that are performed in many developing countries at regular intervals and which provide data for a wide range of monitoring and impact evaluation indicators.

The 2010 Tanzania DHS included a representative probability sample of 10,300 households, which was selected in two stages. In the first stage, 475 clusters were selected from a list of enumeration areas in the 2002 Population and Housing Census. In the second stage, a complete household listing was carried out in all selected clusters between July and August 2009. Households were then systematically selected for participation in the survey.

Three questionnaires were used for the 2010 Tanzania DHS data collection: the Household Questionnaire, the Women’s Questionnaire, and the Men’s Questionnaire. The data utilized for the current study was obtained from the information collected using the women’s questionnaire. This information was collected by interviewing all women aged 15–49 who were either permanent residents in the households included in the 2010 TDHS sample or visitors present in the household on the night before the survey. The women were asked questions on the following topics: Background characteristics (e.g., education, residential history, and media exposure), birth history and childhood mortality, pregnancy, delivery and postnatal care. Also inquired was the information on episodes of childhood illnesses and responses to illness, with a focus on treatment of fevers during the two weeks prior to the survey. The collected information was then transferred into a standard data file, the children’s recode file, which was utilized for the current study. The children’s recode file (KR) defines the unit of analysis as all children born within 5 years period preceding the survey (age 0–59 months). The file is publicly available to researchers free of charge and was obtained by contacting MEASURE DHS [22].

### Wealth Index

The wealth index was constructed using household asset data and principal components analysis [11]. Household asset information included ownership of a number of consumer items, ranging from a television to a bicycle or car, as well as information on dwelling characteristics, such as source of drinking water, type of sanitation facilities, and type of materials used in dwelling construction. Each asset was assigned a weight generated through principal component analysis, and the resulting asset scores were standardized in relation to a standard normal distribution with a mean of 0 and standard deviation of 1 [11]. Each household was then assigned a score for each asset, and the scores were summed for each household. Individuals were ranked according to the total score of the household in which they resided. The sample was then divided into five quintiles from lowest to highest [11]. This was further reduced to three categories by collapsing the upper two and lower two categories.

### Data Analysis

Using the DHS recode manual [23], which lists and describes all variables used in DHS data, we carefully studied the data set and made the necessary categorizations. Analysis was done using SPSS version 19 taking into account the clustering effect. The proportions of children reported with fever, diarrhoea and/or acute respiratory infection (defined by history of cough and difficult in breathing), were studied in relation to caretakers’ background characteristics. Furthermore, we calculated the proportions (with 95% confidence intervals) of caretakers who utilized different types of health care facilities as their first option for child care in relation to background characteristics. Multiple logistic regression analyses were used to further study the observed associations and adjust for potential confounders i.e. residence, socioeconomic status (SES), education and number of living children below five years. Children were categorized as having received proper antimalarials if they had received Quinine, Artesunate or a combination with Artemisinin. Children were categorized as having received old antimalarials if they had received Sulphadoxine-pyrimethamine (Fansider), Chloroquine or Amodiaquine alone. Prompt treatment was defined as having received an antimalarial during the same or next day as the onset of fever.

### Ethics Statement

The data collection procedures for the Demographic and Health Surveys are to a large extent standardized and widely accepted. The broad goals of the exercise are explained to the respondents by fieldworkers during their introduction in the household and written consent is obtained from all participants prior all the data collection procedures.

## Results

Of the 8176 children studied, 4135 (50.8%) were born at home while the rest were born at health care facilities. About half of the children were males and 7667 (93.8%) were still alive at the day of the interview. Of the alive children, 3192 (43.7%) were below 24 months and 7324 (95.5%) were living with the respondent. Out of children that were reported dead, 226 (45%) had died before the age of one month while 459 (90.2%) had died before their second birthday.

The survey interviewed a total of 5519 women. The majority were below 36 years (75%), were married (76.5%) and were from rural areas (77%). Most women had some or completed primary education (54.6%) and were farmers or self employed (65.5%). Seventy eight percent of women had given birth to a total of five or less children. Nearly one in three women (28.7%) had lost at least one child in the past while 476 (8.6%) had lost at least one child below the age of five in five years preceding the survey.

Table 1 shows the prevalences of the three common childhood illnesses (fever, diarrhoea and acute respiratory infection, ARI) versus the background characteristics of the studied women and children. More than a third (2,840, 37.1%) of the studied children were reported having atleast one of the studied illnesses during the two weeks preceding the survey. According to the mothers’ report**,** 1754 children (22.9%) had experienced fever, 1109 (14.9%) had experienced diarrhoea and 589 (7.7%) had experienced cough with short rapid breathing during the two weeks preceding the survey. The proportion of children with fever and diarrhoea was higher in urban areas and among children aged 12–23 months. Fever and diarrhoea was also slightly more commonly reported by women in the higher compared to lower SES group, as well as among those with higher compared to no education.

**Table 1:** Background factors as determinants of occurrence of common illnesses in children under the age of five in Tanzania, 2010.

	Total* (%) n = 7667	Fever (%) n = 1754	OR	Diarrhoea (%) n = 1109	OR	ARI^1^ (%)n = 589	OR
**Residence**							
Urban	1530 (20.0)	454 (29.7)	1.6 (1.2–2.0)	276 (18.0)	1.4 (1.1–1.8)	139 (9.1)	0.9 (0.6–1.2)
Rural	6137 (80.0)	1300 (21.3)	Ref	833 (13.6)	Ref	449 (7.3)	Ref
**Child Age**							
0–11 months	1644 (22.5)	384 (23.3)	1.1 (0.9–1.3)	316 (19.2)	1.9 (1.5–2.3)	148 (9.0)	1.0 (0.7–1.5)
12–23 months	1549 (21.2)	458 (29.6)	1.5 (1.3–1.8)	325 (21.0)	2.1 (1.8–2.5)	144 (9.3)	1.0 (0.7–1.4)
24+ months	4105 (56.3)	900 (21.9)	Ref	457 (11.1)	Ref	291 (7.1)	Ref
**Child sex**							
Males	3810 (49.7)	880 (23.1)	1.0 (0.9–1.2)	579 (15.1)	1.1 (1.0–1.3)	314 (8.2)	1.1 (0.9–1.4)
Females	3857 (50.3)	874 (22.7)	Ref	531 (13.8)	Ref	274 (7.1)	Ref
**SES** 2							
Lower	3437 (44.8)	724 (21.1)	0.7 (0.6–0.9)	470 (13.7)	0.8 (0.7–1.1)	223 (6.5)	1.0 (0.7–1.4)
Middle	1710 (22.3)	360 (21.1)	0.7 (0.6–0.9)	243 (14.2)	0.9 (0.7–1.1)	139 (8.1)	1.1 (0.8–1.7)
Higher	2520 (32.9)	670 (26.6)	Ref	396 (15.7)	Ref	227 (9.0)	Ref
**Caretakers’**							
**education**	1959 (25.6)	445 (22.7)	0.8 (0.5–1.1)	267 (13.6)	0.7 (0.4–1.0)	114 (5.8)	1.1 (0.6–1.9)
No education	5219 (68.1)	1172 (22.5)	0.7 (0.6–1.0)	752 (14.4)	0.7 (0.5–1.0)	435 (8.3)	1.6 (0.6–2.5)
Primary Secondary or higher	489 (6.3)	137 (28.0)	Ref	90 (18.4)	Ref	40 (8.2)	Ref

First column of percentages shows the proportion of total subjects for the various background factors. Other percentages show the proportions of the study subjects with the three illnesses in different categories of the background characteristics.

1 Acute Respiratory Infection.

2 Socioeconomic status.

OR Odds Ratio.

* Different total secondary to missing responses.

More than half of the sick children were taken to a health care provider: 64.3% with fever, 53.3% with diarrhoea and 60.8% with symptoms suggestive of ARI. The most common first option for child care was PHC facilities, followed by private pharmacies. Table 2 shows the proportion of children reported being attended at different types of health care facilities as the first option for the three disease conditions studied.

**Table 2:** First option of care attended for common childhood illnesses in Tanzania.

	Fever (%) (n = 1754)	Diarrhoea (%) (n = 1109)	ARI^2^ (%) (n = 589)
**Place first sought treatment**			
PHC^1^ Facilities	801 (54.0)	448 (59.3)	245 (51.1)
Private pharmacy	344 (23.2)	157 (20.8)	126 (26.3)
Private facility	116 (7.8)	45 (6.0)	33 (6.9)
Higher level hospitals	106 (7.2)	39 (5.2)	40 (8.3)
Religious facility	101 (6.8)	49 (6.4)	32 (6.7)
*Other	14 (1.0)	17 (2.3)	13 (1.0)

Column percentages presented above, showing the proportion of children attended at various health care facilities as the first option of care. Only children of caretakers who sought care outside homes are presented here, hence the total is not 100%.

1 Primary Health Care (Dispensaries or Health Centre).

2 Acute Respiratory Infections.

* Other types of care, not presented above e.g. community health workers, local shops, mobile clinics, NGOs etc.

Only twenty women reported having utilized both PHC facilities and higher level hospitals for the same illness in this study and none of the respondents reported having received traditional care. In total, 115 women (6.6%) reported having utilized higher level hospitals, among which more than 90 percent also reported them being their first option for child care.

### Management of the Common Childhood Illnesses

Among the 1754 children with a history of fever in two weeks preceding the survey, only 284 (26.6%) had received a blood test during the illness. Blood testing was more commonly reported among children attended at non-public compared to public facilities; 71.7% and 81.1% among children solely attended at religious and private facilities respectively compared to 25.6% and 43.1% among children solely attended at PHC facilities and higher level hospitals (district, regional or higher) respectively.

**Figure 1:**
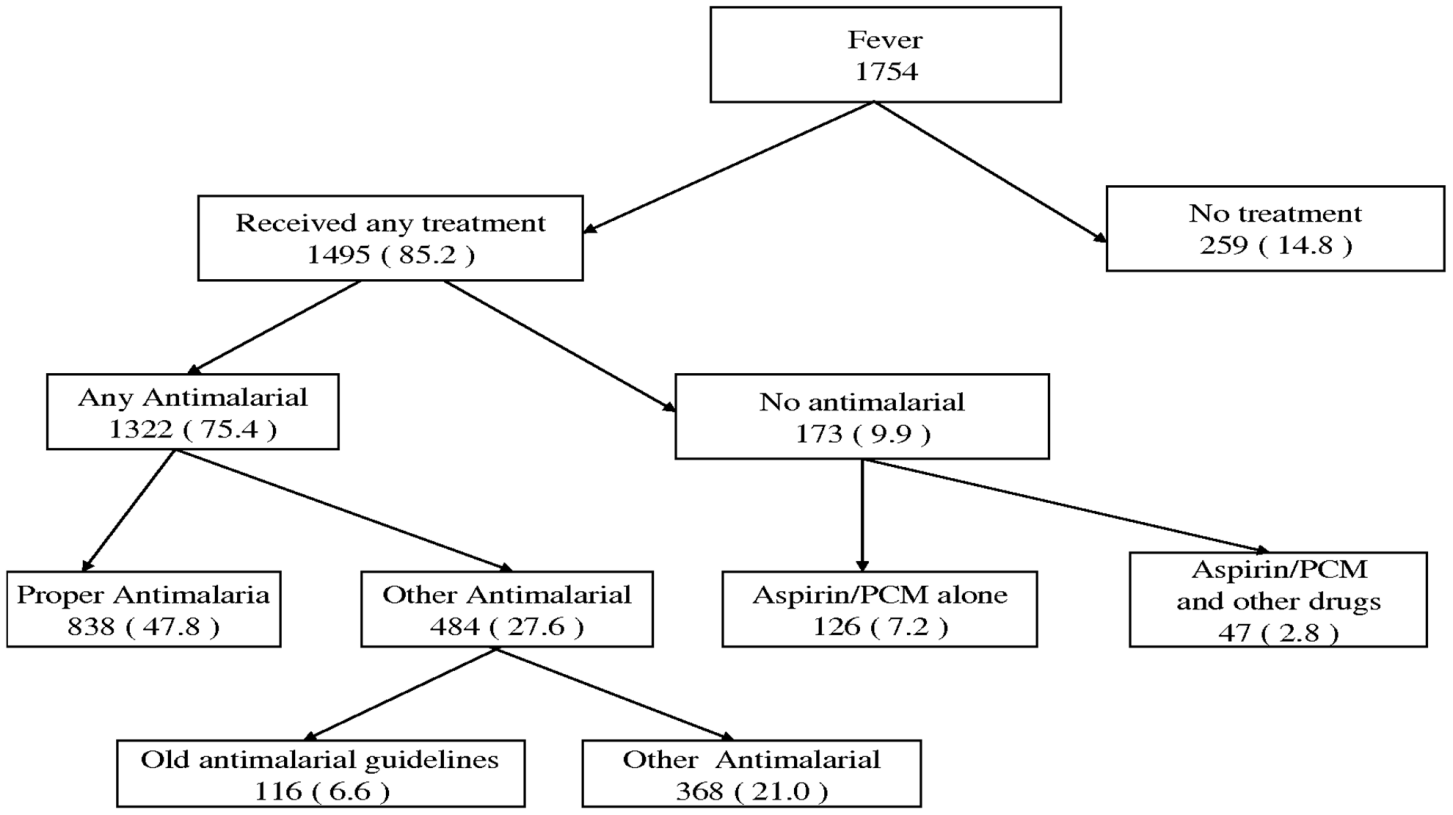
Treatments received for fever as reported by caretakers in Tanzania. • PCM = Paracetamol. • All proportions given above (in brackets), are calculated using all children with fever as a denominator. • Old antimalarial guidelines refer to drugs that are no longer recommended for malarial treatment due to resistance e.g. Fansider, Chloroquine or Amodiaquine.

Figure 1 summarizes the treatments received for fever among our sample of children. Seventy five percent of all children with fever had received any antimalarial. However, only 47.8% had received a proper antimalarial drug as defined by the WHO guidelines for malaria treatment in children. Fifteen percent of children had received no treatment for fever.

Among the children who had received any antimalarial, 36.7% had received the drug the same day, 32.1% on the next day, while the rest had received it two days or later. The proportion of children who received prompt treatment with any antimalarial, i.e. during the same or next day, was found to be higher among children in urban compare to rural areas (OR: 1.5 (1.0–2.4)), among children of mothers with primary or higher compared to no education (OR: 1.3 (0.9–2.0)), and in children of mothers with only one child compared to those with 2 or more children below five years (OR: 2.0 (1.4–2.8)).

Table 3 shows the proportion of children who received proper antimalarials and the promptness of treatment with any antimalarial with respect to the type facility attended for the sick child. For this analysis we only selected children who were reported being attended at only one type of facility. We found that the proportion of children who received proper antimalarials was higher among children solely attended at higher level hospitals (76.5%) followed by PHC facilities (62.1%), and was lowest among children attended at pharmacies (37.9%). On the other hand, the use of drugs according to the old guidelines for malaria treatment was more commonly reported by caretakers solely attended at private facilities (15.6), followed by pharmacies (12.8). Use of old guidelines for malaria treatment was also reported among children solely attended at PHC facilities (5.2%) and higher level hospitals (2.9%). Promptness of treatment with any antimalarial did not differ much with respect to the type of facility consulted (between 47–55%) except for children attended at pharmacies, where only 32.1% had received any antimalarial within the first or second day of onset of fever.

**Table 3:** Only source of care versus timing and proper antimalarial for fever.

Only facility attended	Frequency within fever (%)	Received a proper^1^ antimalarial (%)	Received Old^2^ antimalarial Drug (%)	Prompt^3^ treatment with any antimalarial (%)
PHC facility only	776 (100)	482 (62.1)	40 (5.2)	395 (50.9)
Higher level Hospital only	102 (100)	78 (76.5)	3 (2,9)	56 (54.9)
Pharmacy only	327 (100)	124 (37.9)	42 (12.8)	105 (32.1)
Religious facility only	100 (100)	59 (59.0)	7 (7.0)	47 (47.0)
Private facility only	109 (100)	59 (54.1)	17 (15.6)	54 (49.5)

The table ONLY includes children who were reported being solely attended one type of health care facility (children who were seen at more than one facility are not included). Row percentages are provided here, from the total number of children in the first column.

1 Children are categorized as having received proper antimalarials if they had received Quinine, Artesunate or a combination with Artemisinin.

2 Children are categorized as having received old antimalarials if they had received Fansider (SP), Chloroquine or Amodiaquine alone.

3 Prompt treatment is defined as having received an antimalarial on same or next day after of onset of fever.

**Figure 2:**
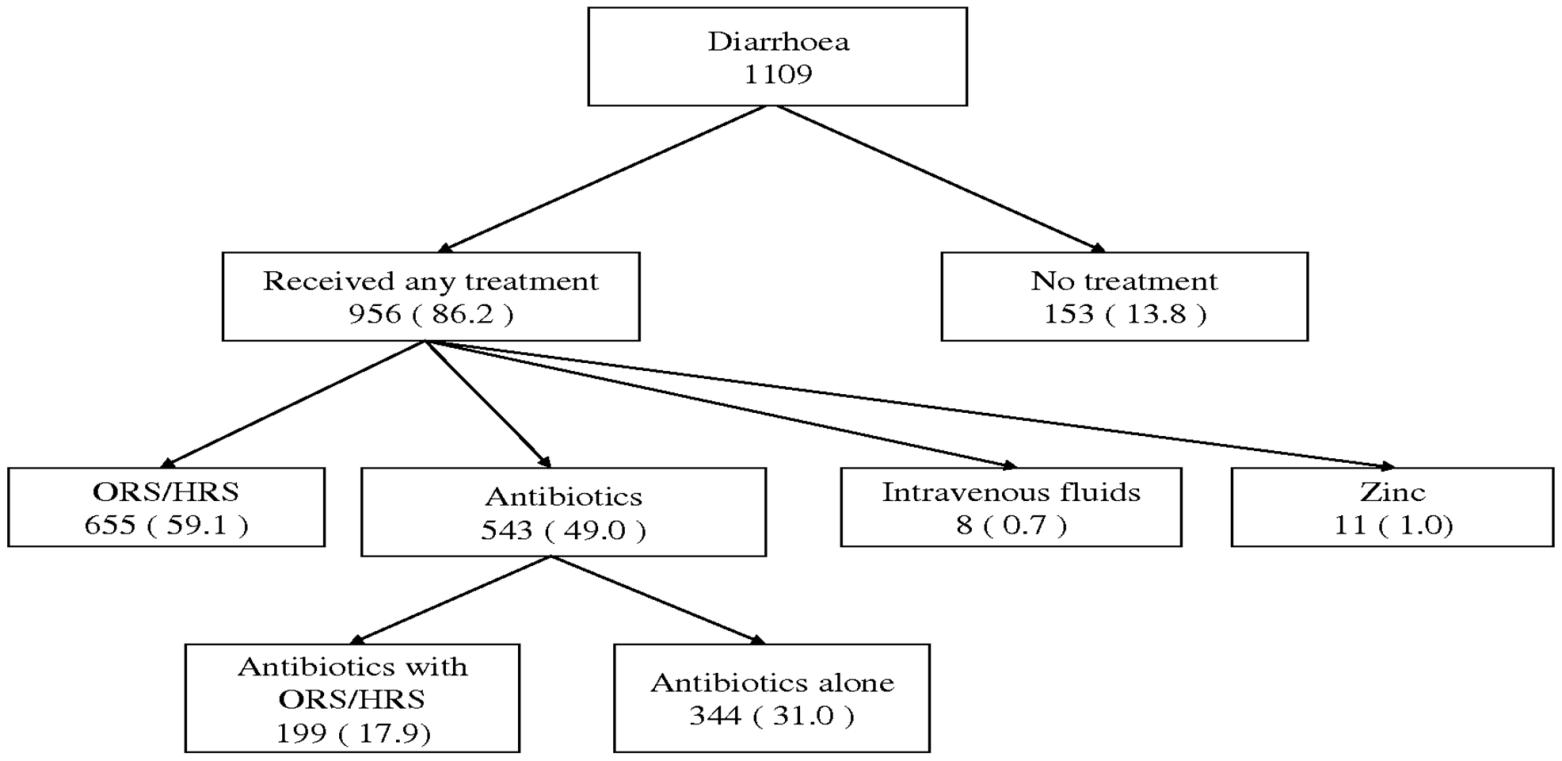
Treatments received for diarrhoea as reported by caretakers in Tanzania. • ORS = Oral Rehydration Salts. • HRS = Home Rehydration Solution. • All proportions given above (in brackets), are calculated using all children with diarrhoea as a denominator.

Figure 2 summarizes the treatments received by children for diarrhoea. Almost 90 percent of all children with diarrhoea had received some treatment. Six in ten children had received oral rehydration salts (ORS) or home rehydration solution (HRS). About half of the children with diarrhoea had received antibiotics; in 31% as the only treatment for diarrhoea. Having received antibiotics for diarrhoea treatment in children was not significantly associated with any of the background characteristics studied. Intravenous fluids and Zinc use for diarrhoea treatment was very low.

The proportion of children receiving ORS/HRS for diarrhoea was highest among those solely attended at higher level hospitals (75.9%) followed by PHC facilities (73.4%) and was lowest among children solely attended at pharmacies (57.5%). The proportion of children who received ORS or HRS was also higher among caretakers with higher compared to lower SES (OR: 1.8 (1.2–2.8)), as well as in children of mothers with only one child compared to those with 2 or more children below five years (OR: 1.4 (1.1–1.9)). Caretakers’ level of education did not significantly influence ORS/HRS use for diarrhoea. We did not have sufficient information to study treatments received for acute respiratory infections.

### Factors Associated with the First Option of Care Attended for Fever and/or Cough

Table 4 and 5 shows associations between background characteristics of the participating women and children in relation to the first option consulted for child care for cough and/or fever. We found PHC facilities utilization as the first option for child care to be more common in rural compared to urban areas as well as among women with a lower compared to higher number of living children below five years. PHC facilities utilization was also found to be more common among women with lower education and SES in the univariate analysis but the association disappeared after adjusting for potential confounders. On the other hand, higher level hospitals were more commonly utilized as the first option for child care by women in urban areas as well as among those with higher level of education. Higher level hospitals utilization was also found to be higher among women in the higher socioeconomic group in the univariate analysis, but this association as well disappeared after adjusting for potential confounders.

**Table 4:** Public facilities as the first option of care for fever and/or cough versus background characteristics.

Background characteristic	PHC facilitiesn = 991% (CI)	AOR^2^	Higher level hospitalsn = 125% (CI)	AOR^2^
**Residence**				
Urban	34.5 (26.8–43.1)	Ref	18.5 (11.5–28.4)	Ref
Rural	61.2 (56.0–66.2)	3.1 (1.8–5.5)*	2.6 (1.7–4.0)	0.1 (0.04.–0.5)*
**Child Age**				
0–11 months	55.4 (47.5–63.1)	Ref	6.6 (4.0–10.8)	Ref
12–23 months	56.7 (50.1–63.1)	1.0 (0.8–1.5)	8.0 (5.0–12.4)	(0.6–2.4)
24+ months	52.1 (46.4–57.7)	0.8 (0.6–1.1)	6.0 (3.8–9.4)	0.9 (0.5–1.6)
**Child sex**				
Male	53.0 (47.3–58.6)	Ref	7.3 (5.0–10.5)	Ref
Female	55.1 (49.7–60.5)	1.1 (0.9–1.5)	6.4 (4.0–10.2)	0.8 (0.5–1.2)
**Caretakers’ Age**				
15–18 yrs	72.5 (54.0–85.5)	Ref	6.7 (1.9–20.8)	Ref
19–35 yrs	52.2 (47.0–57.3)	0.5 (0.2–1.2)	6.5 (4.4–9.4)	(0.3–3.7)
36–49 yrs	58.9 (51.0–66.3)	0.5 (0.2–1.3)	8.5 (5.2–13.7)	2.1 (0.5–8.4)
**SES** 1				
Lower	61.0 (54.6–67.0)	Ref	3.4 (1.4 –8.0)	Ref
Middle	58.0 (49.9–65.8)	0.9 (0.6–1.4)	2.3 (1.1–4.8)	0.8 (0.4–1.5)
Higher	45.5 (38.6–52.5)	0.8 (0.5–1.4)	12.5 (8.6–17.7)	0.7 (0.2–2.8)
**Caretakers’ Education**				
No education	56.8 (48.8–64.5)	Ref	2.3 (1.1–4.8)	Ref
Primary	54.4 (49.0–59.7)	0.9 (0.7–1.3)	7.5 (5.2–10.8)	2.5 (1.3–4.9)*
Secondary or higher	44.8 (35.6–54.4)	1.0 (0.6–1.6)	12.5 (6.4–23.2)	2.0 (0.7–5.6)
**Number of children below five years**				
1 child	58.7 (53.3–63.9)	Ref	8.8 (6.0–12.6)	Ref
2 children	50.8 (43.9–57.6)	0.6 (0.4–0.8)*	5.5 (3.1–9.8)	0.8 (0.4–1.5)
3+ children	46.6 (35.1–58.4)	0.4 (0.3–0.7)*	3.4 (0.9–11.7)	0.7 (0.2–2.8)

* Significant findings.

1 Socio-economic status.

2 Adjusted Odds Ratio for residence, SES, Education and number of living children below five years.

**Table 5:** Non-public facilities as the first option of care for fever and/or cough versus background characteristics.

Background characteristic	Pharmacy n = 443% (CI)	AOR^2^	Relig/private n = 257% (CI)	AOR^2^
**Residence**				
Urban	17.7 (9.5–30.4)	Ref	27.9 (21.4–35.4)	Ref
Rural	26.5 (21.8–31.8)	1.3 (0.5–3.1)	9.0 (6.7–11.8)	0.3 (0.2–0.7)*
**Child Age**				
0–11 months	21.9 (15.6–29.8)	Ref	15.7 (11.8–20.6)	Ref
12–23 months	20.3 (15.2–26.5)	1.0 (0.6–1.6)	13.5 (9.7–18.5)	0.8 (0.5–1.3)
24+ months	27.5 (22.5–33.2)	1.5 (1.1–2.0)*	13.5 (10.4–17.3)	0.9 (0.6–1.3)
**Child sex**				
Male	24.6 (19.4–30.6)	Ref	14.5 (11.3 –18.6)	Ref
Female	23.7 (19.5–28.6)	1.0 (0.7–1.3)	13.5 (10.2–17.6)	0.9 (0.6–1.3)
**Caretakers’ Age**				
15–18 yrs	14.4 (4.7–36.8)	Ref	6.4 (1.7–21.4)	Ref
19–35 yrs	25.1 (20.3–30.5)	1.3 (0.4–4.4)	15.2 (12.3–18.6)	3.1 (0.7–12.2)
36–49 yrs	21.8 (16.1–28.8)	1.1 (0.3–4.0)	10.3 (6.5–16.0)	2.5 (0.5–11.8)
**SES** 1				
Lower	26.6 (21.7–32.2)	Ref	8.5 (5.3–13.3)	Ref
Middle	27.2 (20.5–35.0)	1.0 (0.7–1.6)	11.3 (7.3–17.0)	(0.7–2.4)
Higher	20.2 (13.4–29.3)	1.0 (0.6–2.1)	20.6 (16.4–25.7)	1.2 (0.5–2.8)
**Caretakers’ Education**				
No education	27.2 (20.8–34.8)	Ref	12.6 (8.0–19.4)	Ref
Primary	25.5 (21.0–30.7)	0.9 (0.7–1.3)	11.6 (9.1–14.6)	(0.7–1.2)
Secondary or higher	6.5 (2.5–15.5)	0.2 (0.1–0.6)*	35.2 (25.0–47.0)	1.9 (0.9–4.1)*
**Number of children below five years**				
1 child	15.6 (12.6–19.1)	Ref	15.8 (12.5–19.8)	Ref
2 children	29.8 (23.3–37.3)	(1.5–3.0)*	13.3 (9.8–17.9)	1.1 (0.7–1.6)
3+ children	40.5 (30.1–51.8)	3.3 (2.0–5.4)*	8.3 (4.0–16.4)	0.8 (0.3–1.8)

* Significant findings.

1 Socio-economic status.

2 Adjusted Odds Ratio for residence, SES, Education and number of living children below five years.

Private pharmacies were more commonly utilized as the first option for child care by women with lower compared to higher education, as well as among those having more children below five years under their care. Religious and private health care facilities were more often utilized by women in urban areas as well as those with a higher level of education.

## Discussion

PHC facilities were by far the most commonly reported first option for child care in this study. However further analysis showed that these facilities were more commonly utilized by women in rural compared to urban areas, and among those with a lower compared to a higher number of living children below five years. On the other hand, women in urban areas and those with a higher level of education more commonly utilized higher level hospitals as their first option for child care. If valid, these findings may indicate inequity in quality care access with the better off tending to utilize higher level hospitals which are believed to provide better services. These findings are in line with a hospital-based study conducted in the northeastern Tanzania that established a significantly higher bypassing frequency of PHC facilities among caretakers with a higher level of education in preference for district hospitals while seeking child care [24].

Several previous studies have documented poor quality of services at the primary care level in Tanzania [18], [19], [20], [24], [25], including lack of diagnostic facilities and frequent shortages of drugs and health workers. Lack of diagnostic facilities was found to be the main reason for bypassing PHC facilities for child care in the study mentioned above, conducted in the northeastern Tanzania [24]. Diagnostic tests are crucial in reaching the correct diagnosis in clinical evaluations, and in a country like Tanzania where malaria is the leading cause of morbidity and mortality [11], a blood test to rule out this major childhood killer is important to all children presenting with a history of fever. Findings from a study that evaluated the diagnostic accuracy and case management of clinical malaria in the primary health services in a rural area of south-eastern Tanzania demonstrates the importance of blood testing [15]. In that study, the attending clinicians clinically diagnosed 640 (41.1%) of all consultations as malaria cases while the study showed that only 397 (25.5%) of all consultations were confirmed malaria cases based on a blood slide [15]. Furthermore, 118 (30.2%) of confirmed malaria cases that were misdiagnosed as other infections by the attending clinicians went home without an antimalarial drug prescription while some children who were misdiagnosed as malaria cases by clinicians went home with only an antimalarial.

Realizing the necessity of blood testing in the fight against malaria, the WHO Global Malaria Programme issued in 2010, revised guidelines for the treatment of malaria [26]. In these guidelines, it was recommended that all suspected cases of malaria receive a diagnostic test prior to treatment. The availability of inexpensive, quality assured rapid diagnostic tests for malaria means that parasite-based diagnosis can be achievable even at peripheral health care facilities as well as the community level. However, in the current study, only one in four children with a history of fever had received a blood test, with PHC facilities having the lowest rate of blood testing.

Private pharmacies were the second most common first option for child care in this study, serving nearly one in four children. Private pharmacies are very poorly regulated in Tanzania, and have been reported to sell unregistered, abandoned and sometimes expired drugs [27]. Preference for private pharmacies as the first option for child care has been previously documented in Tanzania [21], [28] **,** as well as in other parts of Sub-Saharan Africa [29], [30], [31], [32]. This practice needs interventions as it could be associated with delays in accessing appropriate treatments and could be contributing to child deaths from treatable illnesses, particularly among children of non-educated families who were more commonly utilizing such facilities in the current study. In the present study, among the 111 children who received old guidelines for malaria treatment, 42 (37.8%) were solely attended at private pharmacies. In line with these findings, another hospital-based study found severe disease to be more common among children who received their first treatment other than paracetamol from private pharmacies compared to those who did not [33]. In that study, private pharmacies were more commonly utilized as the first option for child care by women with lower level of education, as well observed in the current study.

Despite the fact that diarrhoea continues to accounts for many child deaths globally [34], use of the simple and standard treatment for diarrhoea treatment (ORS) remains sub-optimal in many countries, including Tanzania [35]. In the current study, sixty percent of children with diarrhoea had received oral rehydration solution. This is a slight improvement as compared to the 2004–05 Tanzania DHS which reported ORS use among children with diarrhoea to be around 54% [36] but still needs to increase.

Zinc has been proven to significantly reduce morbidity and mortality from diarrhoea in young children [37], [38], [39] and was incorporated in the diarrhoea management guidelines since 2005 [40]. In the current study, extremely few children (<1%) were reported to have received Zinc for diarrhoea treatment. Antibiotics use for diarrhoea treatment is not recommended as it may exacerbate the condition [41], [42], [43]. However, half of children with diarrhoea in this study had received antibiotics and in some cases as the only treatment for diarrhoea. Antibiotics use for diarrhoea treatment in the current study is 10 percent higher compared to the 40 percent reported in the 2004 national survey [36].

Despite the fairly good distribution of health care facilities in Tanzania [12], [13], still half of our studied children were born at home. This poses a danger for the safety of newborns, not only from potential delivery complications but also from the risk of infections secondary to poor delivering environment in most homes. This may be supported by the observation that half of the studied children who were reported dead had died within the first month of life, which could be associated with lack of proper birth care. The observation that nearly a third of all women had experienced at least one child death in the past and one in ten women had lost at least one child below the age of five during the five years period preceding the survey is unacceptable. Furthermore, the finding that 90 percent of all children who died during the five years period preceding the survey had died before their second birthday calls for interventions targeting this age group. In line with the latter findings, several other studies have documented higher child morbidity and mortality during the first year of life [44], [45].

The finding that higher prevalences of fever and diarrhoea were reported by women in urban compared to rural areas and those with higher compared to no education, as well as those with a higher compared to lower SES might be due to reporting bias. These women might be in a better position to remember and report mild cases of such illnesses compared to the non-educated women and women in the lower SES group. We also think the observed positive associations between caretakers’ number of underfives with PHC facilities and private pharmacies utilization may be due to confounding; explained by the fact that caretakers residing in rural areas and those with low education also tend to have a higher number of children.

### Methodological Strengths and Limitations

The current study utilized data from a nationally-representative household survey and hence the findings can be generalized at the country level. It should however be borne in mind that these data are subjective (i.e., based on the mother’s perception of illness) and not validated by a medical examination. The completeness of reporting of the past illnesses is prone to recall bias and may vary between different groups of caretakers in relation to the level of education and SES. Hence, variations in prevalences of illnesses between groups as well as the reported treatments received by sick children should be interpreted with caution. Due to the retrospective nature of this study, we as well did not have sufficient information to answer some of the questions regarding the management of sick children with the three conditions of our interest.

### Conclusions

The management of illnesses accounting for the majority of underfive deaths is still sub-optimal in public as well as the private health care facilities in Tanzania. The observation that PHC facilities and private pharmacies, which tended to offer the poorest services, were more commonly utilized as the first option for child care by women in rural areas as well as those with lower level of education may in part explain the higher child mortality repeatedly reported in these groups. Interventions targeting the quality of care at these facilities are needed to further reduce child mortality from treatable illnesses in Tanzania.
